# Study to determine the safety and efficacy of microneedling as an effective treatment for acne vulgaris

**DOI:** 10.1002/ski2.264

**Published:** 2023-07-05

**Authors:** Mona L. Alqam, Brian C. Jones, Thomas M. Hitchcock

**Affiliations:** ^1^ Crown Laboratories Scientific Affairs Division Dallas Texas USA; ^2^ Crown Laboratories Chief Science Officer Dallas Texas USA

## Abstract

**Background:**

Acne is an inflammatory disease of the pilosebaceous unit that occurs primarily in adolescents. There is no current ideal treatment for acne vulgaris, as many mainstay prescription treatment modalities can compromise the skin microbiome or have deleterious health effects. Further research is needed to investigate novel treatment modalities that account for the importance of the skin microbiome. Other developing treatment modalities for acne are still taking a similar mode of action as current treatments by trying to eliminate *Cutibacterium acnes* despite growing evidence that some *C. acnes* strains may be symbiotic in nature. The perception that microneedling will exacerbate the disease state and trigger more acneic lesions via the spread of acne‐associated microbes has hindered research investigating whether microneedling is a safe and effective treatment. This pilot clinical study challenges such perceptions by clinical assessment to determine if microneedling may produce beneficial treatment outcomes without disrupting critical skin structure or skin microbiome.

**Objectives:**

Test the safety and efficacy of microneedling as an effective treatment modality for acne vulgaris.

**Methods:**

Subjects were split into two groups, one group received three treatments 4 weeks apart, and the second group received four treatments 2 weeks apart. Subjects received an acne assessment by an expert clinical grader at all clinical visits.

**Results:**

There was a statistically significant reduction in both non‐inflammatory and inflammatory lesions at the 2‐month follow‐up compared to the baseline for Group 1. Group 1 and Group 2 saw a decline of 48.20% and 54.00% in non‐inflammatory lesions and 57.97% and 36.67% in inflammatory lesions, respectively, at their last visit compared to baseline.

**Conclusion:**

This study expands the utility of microneedling into a potential therapeutic modality for acne vulgaris. The data generated during the duration of this clinical study demonstrates that there is no scientific reason for microneedling to be contraindicated for acne. In this pilot, microneedling did not cause post‐treatment complications and was seen to reduce acne lesions effectively. Thus, microneedling may have the potential to be a well‐tolerated option for those suffering from acne, being a treatment that neither damages the sebaceous glands nor disrupts the skin microbiome.

## Graphical abstract

Acne is an inflammatory disease of the pilosebaceous unit that can occur at any point in life. Currently, no ideal treatment exists as many mainstay prescription treatment modalities can compromise the skin microbiome or have harmful health effects. The data generated during the duration of this clinical study demonstrates that microneedling may have the potential to be a well‐tolerated option for those suffering from acne, being a treatment that neither damages the sebaceous glands nor disrupts the skin microbiome.

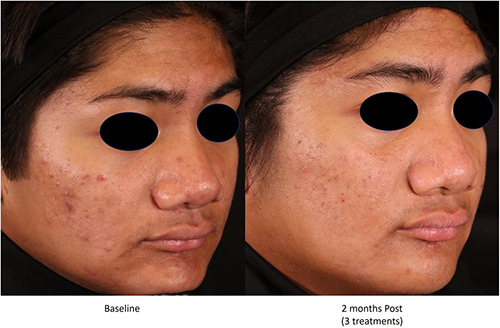

**What is already known about this topic?**

- Acne is a common inflammatory disease that primarily affects adolescents. There is a need for non‐pharmacological treatments for acne that do not compromise the skin microbiome or damage the sebaceous glands. To date, no one has investigated if microneedling is an effective treatment for acne. Yet, it is widely accepted that microneedling is not an acceptable treatment modality for acne as it would further exacerbate the disease state.

**What does this study add?**

- This study discovers a new effective and safe treatment modality to reduce the number of inflammatory and non‐inflammatory acneic lesions. Provides empirical evidence that does not support the claim that microneedling would cause more acneic flare‐ups, but rather produces beneficial treatment outcomes.

## 1 INTRODUCTION

Acne vulgaris, commonly referred to as acne, is a prevalent skin disorder affecting the pilosebaceous unit of the skin, impacting nearly all individuals at some point in their lives.^1^ Acne occurs most frequently in adolescence but can persist well into adulthood. Multiple factors are thought to contribute to acne development, such as increased sebum production, hyperkeratinization, colonization, and hyperproliferation of specific bacterial or fungal species strains within follicles.^1^ It is widely accepted that these phenomena can lead to either inflammatory lesions (papule, pustule, and nodule) or non‐inflammatory lesions (open and closed comedones) when sebaceous follicles are blocked by sebum and dead cells.^1^ Additionally, a repercussion of acne can be permanent facial scarring.^2^

A reductive model of acne that seems to persist is that increased sebum production causes the over‐proliferation of the bacterial species *Cutibacterium acnes (C*. *acnes)* in follicles and induces an inflammatory response.^3^ However, the advent of metagenomic sequencing has shown that *C*. *acnes* is one the most abundant and widely distributed bacteria on healthy skin.^3^, ^4^ The abundance of *C*. *acne*s on healthy skin suggests that the involvement of the *C*. *acne*s species in the pathology of acne is strain‐specific, and does not necessarily implicate the entire species. Further, new lines of evidence have demonstrated that the follicles of acneic skin have higher rates of microbial diversity and less *C*. *acnes* species abundance when compared to healthy skin that is predominantly colonized by *C*. *acnes*.^3^, ^5^ It has previously been reported that beneficial strains of *C*. *acnes* can prevent the colonization of bacterial and fungal pathogens (e.g. *Staphylococcus aureus* and *Streptococcus pyogenes*) by producing free fatty acids, short‐chain fatty acids, and antimicrobial agents that produce a skin environment that is not conducive to the growth of pathogenic microbes.^6^ Additionally, recent research has shown that certain strains of *C*. *acnes* are healthy for skin and can contribute to benefits such as antioxidant activity, anti‐inflammatory activity, and upregulation of genes involved in skin barrier homeostasis.^7^

Despite the emerging evidence to the contrary and the ubiquity of *C*. *acnes* on healthy skin, many still refer to acne specifically as a *C*. *acnes‐related infection* of the pilosebaceous unit.^3^, ^8^, ^9^, ^10^ Because of this, first line prescriptive treatments for acne have traditionally included antibiotics. However, antibiotics can also indiscriminately kill beneficial bacteria needed for a healthy microbiome and lead to antibiotic‐resistant bacterial strains.^11^ Another treatment modality for acne is to reduce or damage the sebaceous glands chemically or thermally to downregulate sebum production.^12^ This treatment aims to prevent bacteria from over proliferating in the pilosebaceous unit by depriving them of sebum, their preferred food source on the skin. Significant reduction in sebum is known to dry out skin, lips, and eyes and can have long‐term deleterious health effects.^13^ Retinoids are known for their effectiveness against acne by decreasing the sebaceous gland size in the skin and are a mainstay acne treatment.^14^, ^15^ The long‐term side effects of reducing sebum production by isotretinoin treatment have been correlated with dry skin, dry eye syndrome, skin barrier dysfunction leading towards eczema, and secondary skin infections by *S*. *aureus*.^15^, ^16^ While it is commonly believed that sebaceous glands can recover after being downregulated, previous studies have demonstrated that sebum production is still inhibited years after retinoid treatments such as isotretinoin (Accutane).^15^, ^16^ These methodologies again assume that the overall reduction of the *C*. *acnes* species will reduce pathology but do not consider the long‐term effects of deprivation of the skin biome of potential protective benefits conferred by healthy strains of the species.^7^, ^17^

Additionally, acne vulgaris treatment modalities can be over‐the‐counter or prescription strength in topical or oral therapies that include benzoyl peroxide, azelaic acid, salicylic acid, and oral isotretinoin or as procedures using a medical device that delivers thermal energy to sebaceous glands.^18^, ^19^ These standard treatments to target acne may not be effective for everyone, and some patients do not qualify to take isotretinoin or antibiotics due to skin tone, age, or an underlying medical condition such as pregnancy, photosensitivity, or suicidal ideation. Many patients want to avoid the potential long‐term adverse physiological side effects of systemic isotretinoin, or the possibility of teratogenicity in female patients of childbearing potential.^20^, ^21^ Initial evidence from some pregnancy prevention programs to stop fetal exposures to oral isotretinoin such as iPLEDGE used in the USA has not completely stop expsoure.^22^

Those that suffer from acne can also suffer from depression and social anxiety, and their psychological stress could further exacerbate their acne disease state. Acne sufferers self‐report a poor quality of life as many have low self‐esteem and poor self‐image, with a recent study finding that between 9% and 15% of U.S. acne sufferers are at high risk of suicide.^23^ Oral isotretinoin would not be a recommended treatment for some of these acne patients as isotretinoin has frequently been linked with psychiatric adverse events and has a black box warning for suicide, depression, aggression, and psychosis. With the reduction in the usage of antibiotics due to increasing antibiotic resistance, there is a need for novel acne treatment methods that neither damage the sebaceous glands nor hurt the skin microbiome.^11^, ^18^ Yet, despite all the aforementioned, acne treatment strategies have generally remained unchanged.

Mechanical skin remodeling via the use of microneedles, commonly referred to as “microneedling,” is a well‐studied, safe, and effective skin treatment involving puncturing the epidermis with fine needles to induce a wound‐healing response and subsequent remodeling of skin. The efficacy of treating acne scars and wrinkles has previously been established, and other possible clinical applications for microneedling are being investigated.^24^ Currently, microneedling is contraindicated for treating acne vulgaris in some regions, one being the United States. It is a general perception in the medical community that microneedling over inflammatory acne lesions can act to exacerbate acne via the spreading of *C*. *acnes* bacterium from acneic lesions to other areas of the face. Despite there being no definitive evidence that acne is in any way communicable via microbial transference or that microbial over‐proliferation is connected to developing acne or the severity of the disease state, these ideas seem to persist.^7^, ^25^, ^26^, ^27^, ^28^, ^29^ The common misperception that C. *acnes* is solely responsible for acne or that microneedling could spread *C*. *acnes* across the face has likely hindered research into investigating such as a potential treatment for acne. However, as acne is a multi‐factorial disease state involving the hyperkeratinization of the stratum corneum, which blocks the normal flow of sebum from the follicle, it makes sense that initiation of local skin remodeling via transdermal microneedling (involving rapid turnover of the epidermis and stratum corneum) would allow for relief of acneic symptoms during the remodeling process.

A literature search has shown that no published studies have investigated microneedling as a treatment modality for treating acneic lesions. The empirical data generated in this clinical study aims to show that microneedling over acneic inflammatory lesions does not cause post‐treatment complications or induce acne exacerbation. In fact, microneedling with the proper device and protocols may significantly reduce inflammatory and non‐inflammatory acneic lesions and provides a beneficial treatment outcome.

## 2 METHODS

We screened multiple subjects based on the IRB approved protocol. The investigator reviewed inclusion and exclusion criteria to assess eligibility after obtaining signed informed consent and photo release waivers. The study included subjects that had an Investigator's Global Assessment (IGA) score of 1, 2, or 3 and excluded an IGA score of 4. Patients with a history of inflammatory skin disease other than acne vulgaris, a history of using systemic retinoids in the past 6 months, a history of using topical acne treatments such as anti‐inflammatory agents or antibiotics, and individuals who had cosmetic treatments in the treatment area in the past one to 2 years, and pregnant or nursing women were excluded as well. A total of 15 subjects (11 females and 4 males) who had a clinical diagnosis of acne vulgaris, met inclusion criteria, and were willing to withhold any aesthetic procedure to the face were enrolled in the clinical study. Subjects were assessed by an expert clinical grader at baseline that included an acne assessment by conducting an acne lesion count, assigning an IGA score, and participating in facial photography.

Patients were placed into two groups receiving microneedling treatment using an FDA‐cleared, automated non‐surgical microneedling pen (SkinPen® Precision System; Crown Aesthetics, Dallas, TX). Group 1 (*N* = 9) received three treatments at 4‐week intervals, and Group 2 (*N* = 3) received four treatments at 2‐week intervals. All subjects returned for a 2‐month follow‐up visit post final microneedling treatment for a final acne assessment. Subjects completed satisfaction questionnaires during their last two visits to assess their self‐improvement after receiving multiple treatments. Facial images were taken using the default camera settings at all visits pre, and post‐treatment using a Canon EOS 80D (Tokyo, Japan). If applicable, a pregnancy test was administered to subjects prior to treatment. Before performing any study‐related procedures, patients had to remove makeup for at least 30 min and acclimate to ambient temperature and humidity conditions. An expert clinical grader used the DermaLab Combo, (Cortex Technology, Denmark) to measure subject erythema prior to all microneedling treatments. A prescription compounded numbing cream (20% Benzocaine, 6% Lidocaine, and 4% Tetracaine) was applied to the subject's face to make the microneedling procedure more tolerable. A clinician removed excess numbing cream from the treatment area with gauze. After 20 min, the numbing cream was removed by applying a facial cleanser (SkinFuse Purify Cleansing Complex, SkinFuse®; Crown Aesthetics, Dallas, TX) to the face and gently messaging the cleanser into the skin and wiping the cleanser off with water and a damp gauze. Multiple passages were made over the subject's face with gauze to ensure the subject's face was cleansed and dry. A non‐medicated hydrogel wound dressing was applied to the subject's treatment area to protect against abrasion and friction during the microneedling procedure (SkinFuse® Lift HG; Crown Aesthetics, Dallas, TX). A clinician would treat each subject's face from hairline to jawline with the microneedling pen with treatment depths of up to 2.5 mm. After three passes to each treatment area with the microneedling device, the clinician would return and use the stamping technique on papular and pustular lesions. The clinician used the stamping technique to induce pinpoint bleeding on papules and until pustular content was released from the lesion. Treatment depth only exceeded 2.0 mm when the clinicians performed the stamping technique on inflammatory lesions. Each subject had the microneedling treatment depth recorded at every visit. Subjects received a SkinFuse® skincare regimen, including cleanser, sunscreen, moisturizer, and usage instructions to maintain skincare product consistency amongst all subjects (SkinFuse®; Crown Aesthetics, Dallas, TX). Subjects received a diary to record any adverse events between visits.

At all subsequent visits, an expert clinical grader recorded concomitant medications and asked if subjects had experienced any changes in their health since the previous visit. Acne lesion count and IGA assessment were completed before every treatment. An expert clinical grader assessed acne lesions by counting the number of inflammatory lesions, including papules, pustules, nodules, and non‐inflammatory open and closed comedones.

## 3 RESULTS

**FIGURE 1:**
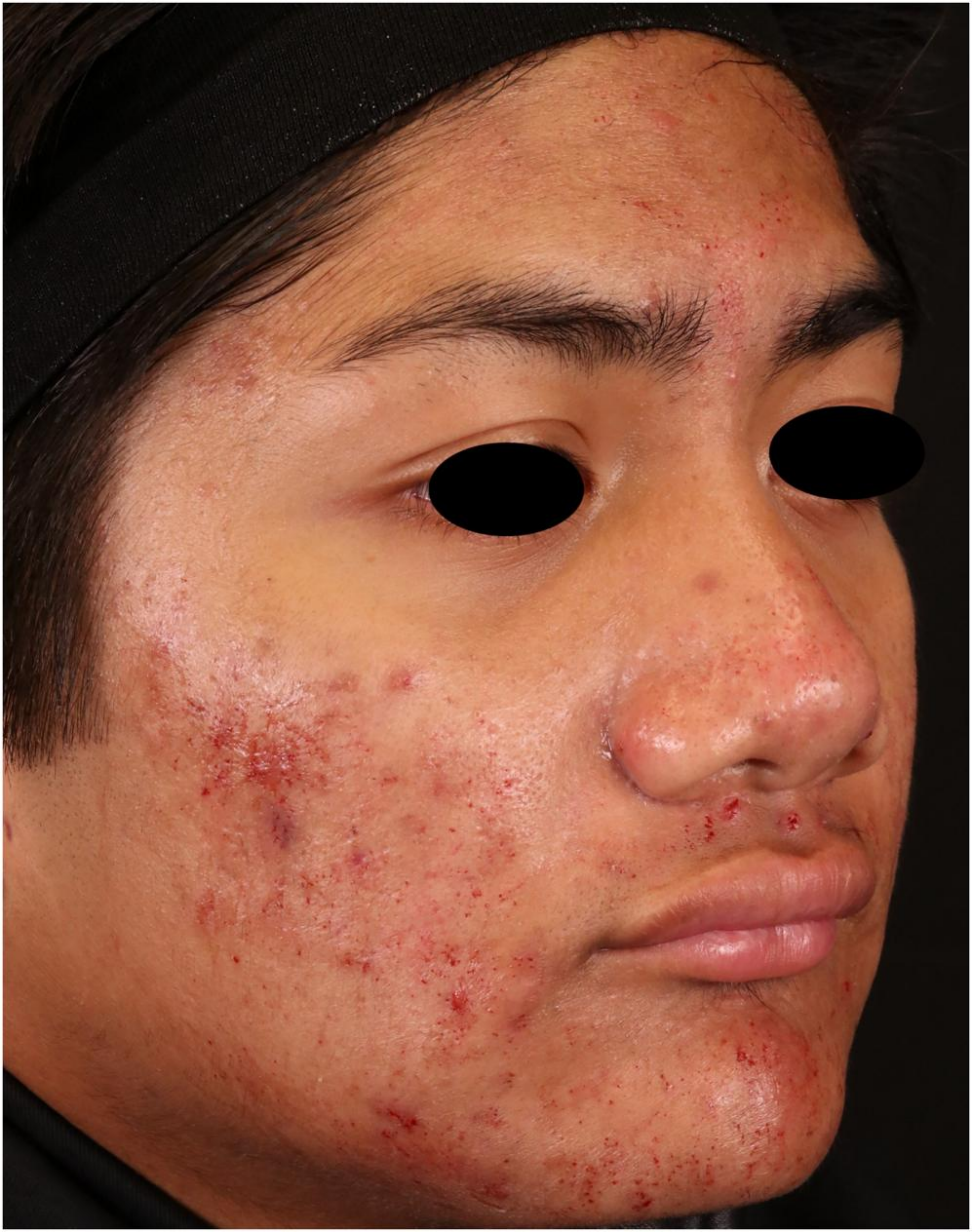
Subject side view image post‐treatment. The subject received three passes with the microneedling device. The stamping technique was used over inflammatory lesions with a depth of 2.0–2.5 mm in order to induce pinpoint bleeding on papules and to release the pustular content.

**FIGURE 2:**
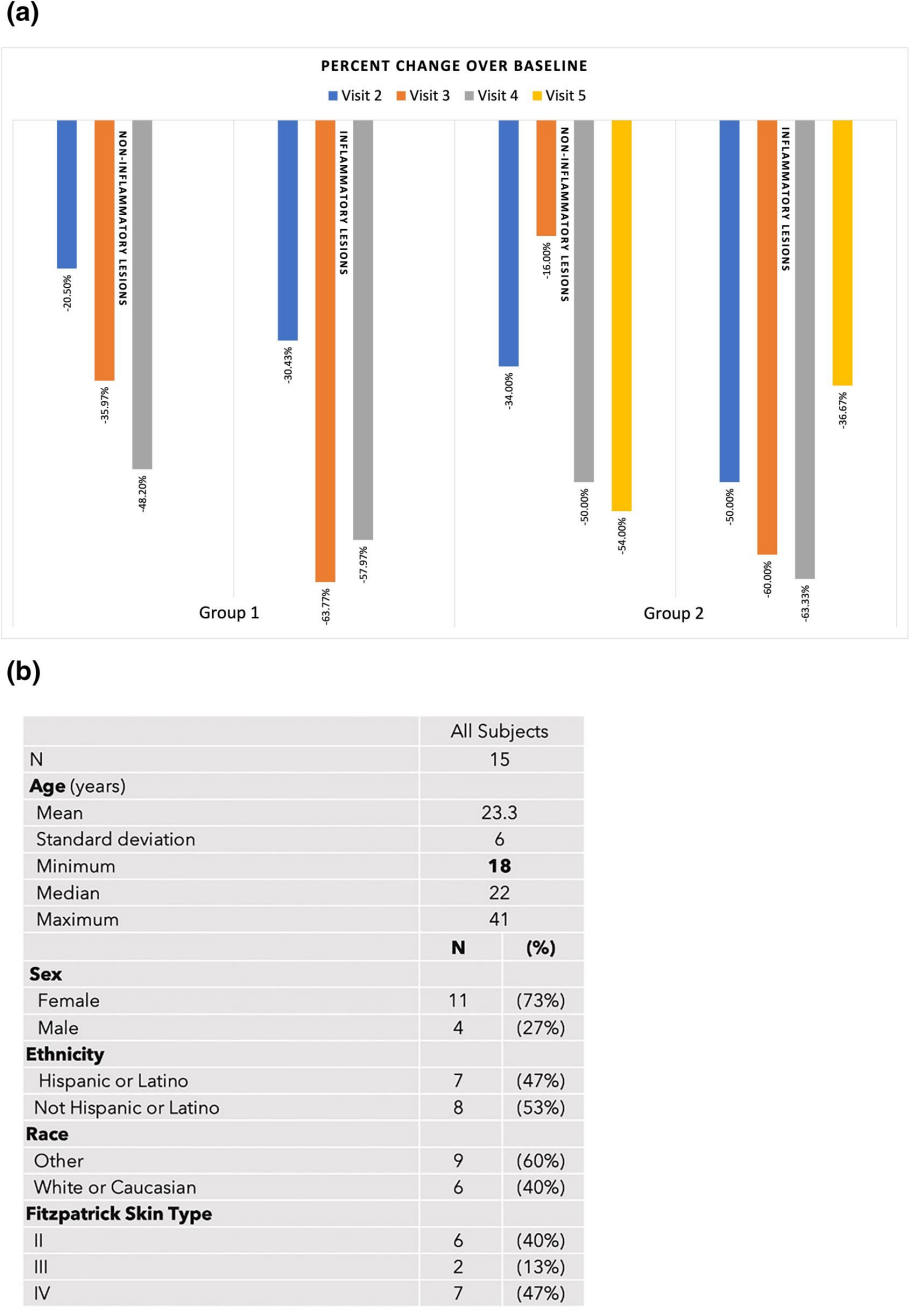
(a). The percent decrease in both non‐inflammatory and inflammatory acne lesions for both Group 1 and 2 at each time point against baseline. (b). Demographic data from subjects that participated in our clinical study to investigate the efficacy of microneedling to treat acne vulgaris.

**FIGURE 3:**
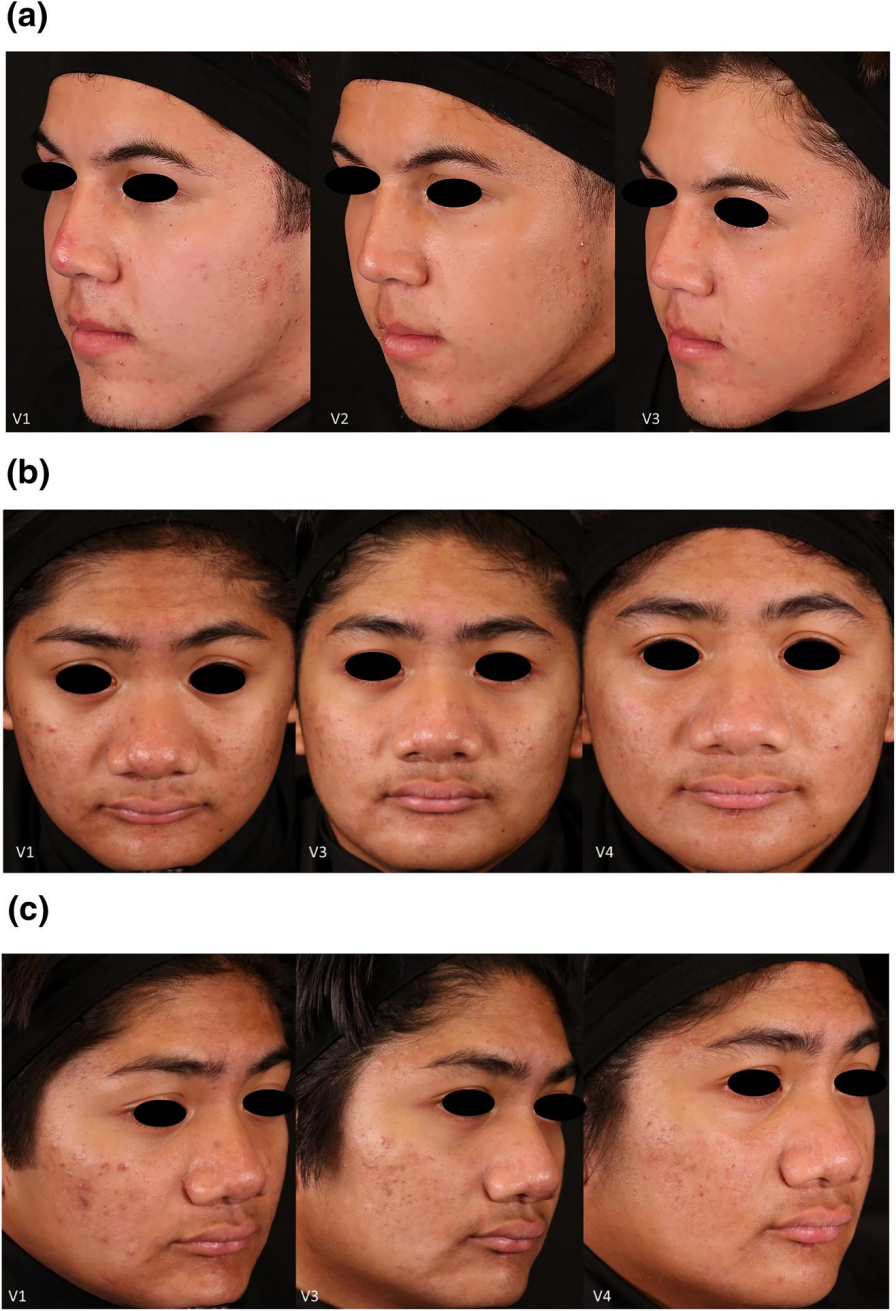
(a) 20‐year‐old male in Group 1 with acne. Visit 3 is after 2 microneedling treatments. Subject had a significant decrease in the number of inflammatory lesions and a two‐grade decrease on the Investigator's Global Assessment (IGA) scale (b) Front and (c) side view: A 19‐year‐old male in Group 1 with Fitzpatrick skin type III. Visit 3 (V3) is after 2 microneedling treatments. Visit 4 (V4) is the 2 month follow up visit. These treatments were spaced 4 weeks apart. A clinically significant reduction in inflammatory and non‐inflammatory lesions was observed.

**FIGURE 4:**
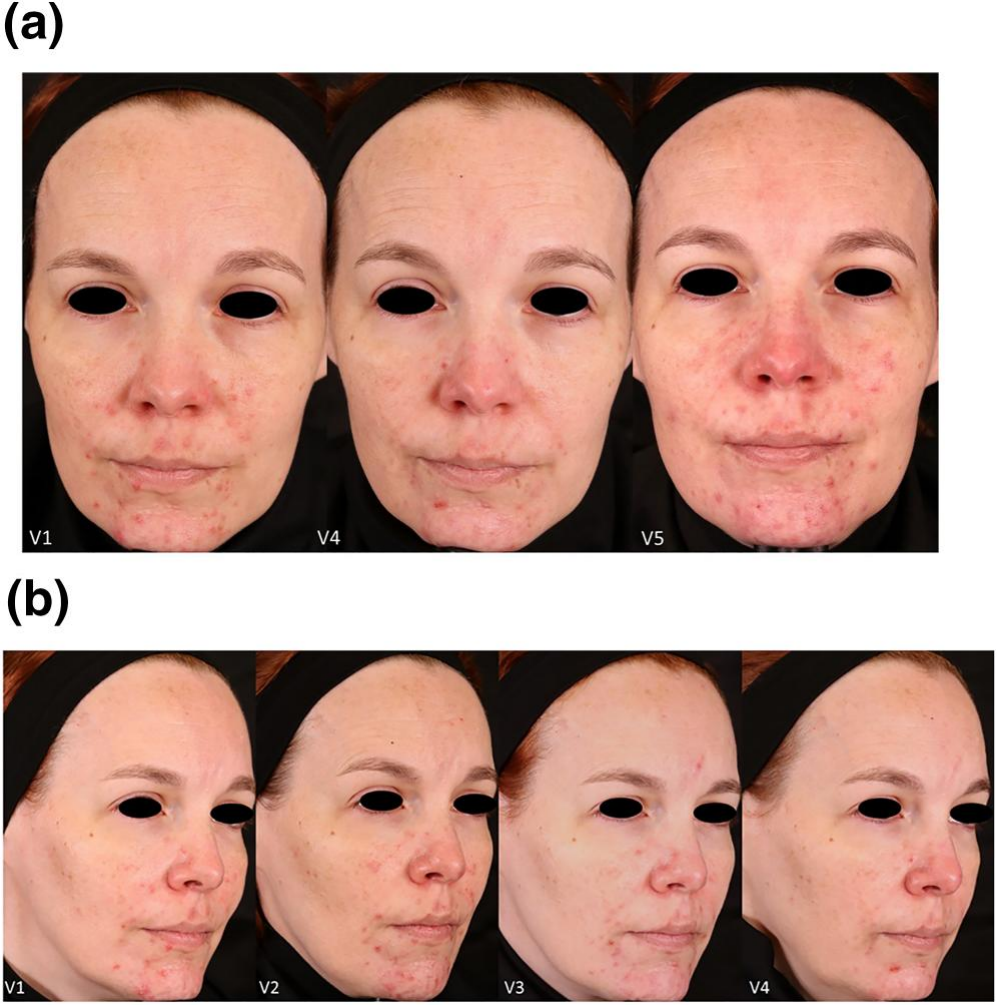
(a). A Caucasian 41‐year‐old female in Group 2, front view. Image at Visit 4 (V4) she had already received 3 microneedling treatments and Visit 5 (V5) is the 2‐month post treatment. Note that the subject had relapsed at Visit 5 as she was in the follow up phase, not receiving microneedling treatments. (b). Side view, visible improvement during active treatment and the significant clinical improvement in her acneic lesion count and a two‐grade reduction on the IGA scale. Visit 4 (V4) is after 3 microneedling treatments, 2 weeks apart.

**FIGURE 5:**
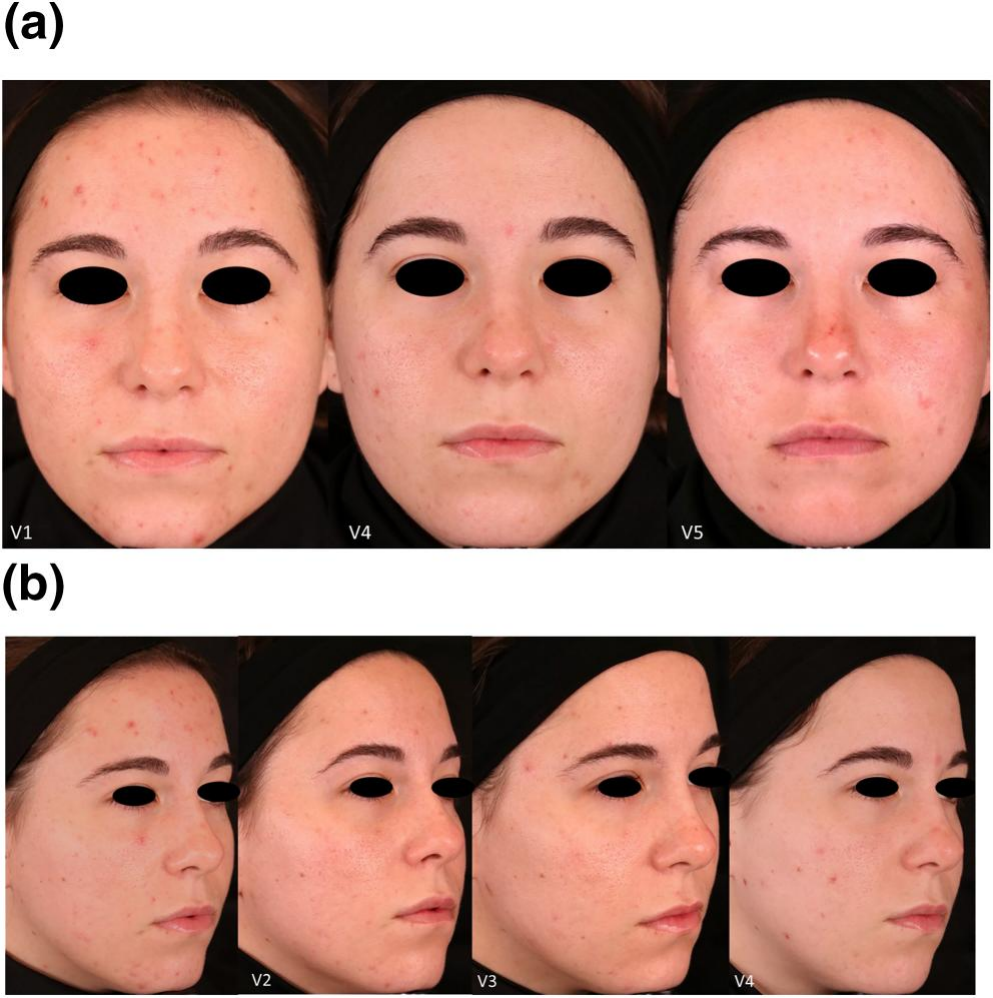
(a). This 25‐year‐old subject in Group 2 had moderate inflammatory lesions at Baseline. A clinical improvement is demonstrated here after 4 microneedling treatments spaced 2 weeks apart. Visit 5 (V5) is the 2‐month post treatment visit. (b). Visit 4 (V4) is after subject received 3 microneedling treatments, 2 weeks apart.

Twelve patients with Fitzpatrick Skin types II‐IV, whose ages ranged from 18 to 45, completed the study. Three subjects discontinued, two for non‐compliance, and one withdrew consent. Figure 1 shows a subject post‐treatment where the stamping technique was used on inflammatory lesions. The percent decrease in acneic lesions at their 2‐month post‐treatment follow‐up (visit 4 for Group 1 and visit 5 for Group 2) was 48.20% and 54.00% in non‐inflammatory lesions and 57.97% and 36.67% in inflammatory lesions when compared to baseline. Figure 2 shows the mean percentage decrease for non‐inflammatory and inflammatory acne lesions at each time point compared to the baseline and demographical data from participants. There was a statistically significant reduction in both non‐inflammatory and inflammatory lesions at the 2‐month follow‐up compared to the baseline for Group 1 (*p*‐values were ≤0.05). Figure 3 has subjects' facial images for subjects in Group 1 that received three microneedling treatments at visit 1 (baseline), visit 2, and visit 3. Statistical significance was not found in Group 2 due to the small sample size. However, after combining the dataset from both groups, we found a statistically significant reduction in both non‐inflammatory and inflammatory lesions (*p*‐values ≤0.05). Figures 4 and 5 have facial images of subjects in Group 2 that received four microneedling treatments at visit 1 (baseline), visit 2, visit 3, and visit 4, showing a reduction in acneic lesions. The mean IGA score at baseline for Groups 1 and 2 was 2.11 and 2.33, respectively. A decline in mean IGA scores was observed in both groups at the 2‐month follow up visit, Group 1 was 1.11 and Group 2 was 1.33. Erythema was measured at all visits, and no changes occurred in erythema during the duration of the study for any subject, further indicating that the procedure was safe to be performed on inflammatory skin.

In assessing the experience and outcome of the treatment, all patients would recommend microneedling as a treatment method for Acne Vulgaris. All but one subject saw a 50% or greater improvement in their acne, with one only seeing a 25% improvement. When subjects were asked how they would characterize their satisfaction with microneedling treatment for acne, 100% stated they were satisfied at Visit 3, 92% at Visit 4%, and 100% at Visit 5.

## 4 DISCUSSION

Skin remodeling via the use of transdermal microneedling procedures has been shown to reduce fine lines and wrinkles, improve skin texture, and improve the appearance of dermatological imperfections such as acne scars, surgical scars, cellulite, and stretch marks.^24^ This study expands the utility of microneedling into a potential treatment modality for acne. The results from this study refute the misconceptions that microneedling exacerbates acne vulgaris and spreads inflammatory lesions across the face and provides data to support microneedling as a safe and effective treatment modality for acne vulgaris. The FDA cleared microneedling device used in this study delivered the needles at precise depths in the treatment area (SkinPen® Precision System; Crown Aesthetics, Dallas, TX) allowing for an effective treatment that does not damage the sebaceous glands, unlike drugs and devices whose mechanism of action is to diminish or impair these important follicle structures. The controlled puncturing of acneic lesions with fine needles to induce a normal wound healing process where a short initial inflammatory period is followed by rapid turnover of the stratum corneum and epidermis, down‐regulation of pro‐inflammatory cytokines, and subsequent remodeling of new dermal tissues is the proposed cascade of events that lead to the observed clearing of acneic lesions in this study. Blockage of sebaceous glands ducts by over‐proliferating keratinocytes will induce acne. It has been previously proposed that introducing Matrix‐Metallo‐Proteinases by microneedles can downregulate keratinocytes hyperproliferation, equilibrate cell proliferation, and thus prevent the blockage of sebaceous glands ducts.^30^ However, additional studies are needed to understand how microneedling clears acneic skin.

Despite the lack of published evidence, it is widely accepted and held as a scientific fact that microneedling is an unacceptable treatment for acne. Misconceptions and warnings about microneedling over acneic lesions are currently being propagated over social media, blogs, and other non‐scientific outlets, despite them providing no empirical data to support such claims.

In this study, subjects in both groups had a decrease in both inflammatory and non‐inflammatory lesions regardless of the time between the treatments. The treatment plan for Group 2 subjects that received a microneedling treatment every 2 weeks was to be more targeted and capture those subjects during an active acne flare‐up state where hormonal changes could exacerbate the disease state. It is conceded that the small sample size of Group 2 limited our statistical power; however, given that Group 1 had a less frequent treatment plan (treatment every 4 weeks) and still had a statistically significant reduction in acneic lesions, we speculate that microneedling more frequently will have an equal or greater reduction of acneic lesions.

The data generated from this clinical study provide objective evidence that does not support the idea that microneedling cannot be used on acneic skin, as our findings demonstrate that there is no scientific basis for microneedling to be contraindicated for treating acne, as is the case currently with marketed mironeedling medical devices. Instead, it may be a valued tool and potentially be a safer alternative for the effective treatment of acne vulgaris. This clinical study could aspire new discussion in the medical community about the current standard of care for acne vulgaris. As we further incorporate our knowledge of the importance of the human skin biome's ecosystem, including health‐associated *C*. *acnes* strains our treatments methods should evolve as new evidence comes out.

The growing acceptance of the microbiome's importance and its role in skin health and the overall well‐being of individuals has only recently begun being appreciated and considered when treating patients.^31^ Traditional treatments that include antibiotics and retinoids can be harsh on the skin microbiome and impair skin barrier function.^32^ In theory, any procedure using a medical device to treat acne by delivering heat to destroy sebaceous glands or a drug to mitigate their function could negatively alter the microbiome's composition. There is a need for non‐pharmacological treatments for acne that do not compromise the skin microbiome or damage the sebaceous glands. Further investigation and a more in‐depth look at how a medical device procedure could impact the skin microbiome is essential as the skin microbiome becomes a novel focus in the treatment of dermatological conditions. Microneedling provides a simple treatment plan with a short recovery time that, in this study, effectively reduces acneic lesions. The ease of microneedling could significantly improve adherence to acne treatment and thus greatly improve treatment outcomes. This study is limited in subject size, and additional clinical studies are necessary to determine the long‐term‐ benefits and efficacy and the possible benefit of utilizing traditional acne topical agents as a maintenance program after discontinuing microneedling or along with microneedling to maintain clear skin. Overall, microneedling demonstrates an effective treatment plan for acne vulgaris.

## CONFLICT OF INTEREST STATEMENT

Mona L. Alqam MD, Brian C. Jones PhD, and Thomas M. Hitchcock PhD are employees of Crown Laboratories, which funded the study presented in the manuscript.

## AUTHOR CONTRIBUTION

**Mona L. Alqam**: Conceptualization (equal); Investigation (equal); Methodology (equal); Project administration (equal); Writing – review & editing (equal). **Brian C. Jones**: Conceptualization (equal); Funding acquisition (equal); Methodology (equal); Project administration (equal); Writing – review & editing (equal). **Thomas M. Hitchcock**: Conceptualization (equal); Funding acquisition (equal); Project administration (equal); Writing – review & editing (equal).

## ETHICS STATEMENT

The authors confirm that the ethical policies of the journal, as noted on the journal's author guidelines page, have been adhered to. Our protocol to use human subjects for our original study was approved by our Institutional Review Board (IRB) for the clinical portion of the study to use human subjects under IRB study number CL‐AC‐21‐02.

## Data Availability

The data will not be made publicly available.
